# Nanoparticles-Based Strategies to Improve the Delivery of Therapeutic Small Interfering RNA in Precision Oncology

**DOI:** 10.3390/pharmaceutics14081586

**Published:** 2022-07-29

**Authors:** Jinxing Huang, Kai Xiao

**Affiliations:** 1 Precision Medicine Research Center, Sichuan Provincial Key Laboratory of Precision Medicine, National Clinical Research Center for Geriatrics, West China Hospital, Sichuan University, Chengdu 610041, China; hesterjx@163.com; 2 Frontiers Science Center for Disease-Related Molecular Network, West China Hospital, Sichuan University, Chengdu 610041, China

**Keywords:** small interfering RNA, nanoparticles, gene delivery, cancer, targeting, combination strategies

## Abstract

Small interfering RNA (siRNA) can selectively suppress the expression of disease-causing genes, holding great promise in the treatment of human diseases, including malignant cancers. In recent years, with the development of chemical modification and delivery technology, several siRNA-based therapeutic drugs have been approved for the treatment of non-cancerous liver diseases. Nevertheless, the clinical development of siRNA-based cancer therapeutics remains a major translational challenge. The main obstacles of siRNA therapeutics in oncology include both extracellular and intracellular barriers, such as instability under physiological conditions, insufficient tumor targeting and permeability (particularly for extrahepatic tumors), off-target effects, poor cellular uptake, and inefficient endosomal escape. The development of clinically suitable and effective siRNA delivery systems is expected to overcome these challenges. Herein, we mainly discuss recent strategies to improve the delivery and efficacy of therapeutic siRNA in cancer, including the application of non-viral nanoparticle-based carriers, the selection of target genes for therapeutic silencing, and the combination with other therapeutic modalities. In addition, we also provide an outlook on the ongoing challenges and possible future developments of siRNA-based cancer therapeutics during clinical translation.

## 1. Introduction

RNA interference (RNAi) is an evolutionary-conserved mechanism that degrades homologous target genes via double-stranded RNA (dsRNA) [1]. Small interfering RNA (siRNA) therapy belongs to RNAi technology, which silences the target messenger RNA (mRNA) through the formation of RNA-induced silencing complex (RISC) by double-stranded RNA (dsRNA) and argonaut 2 helicase in the cytoplasm to block the translation of corresponding protein [2]. Theoretically, siRNA could silence any specific disease-related genes in the way described above. Therefore, siRNA has been recognized as an indispensable tool to study the function of single genes and a new potential therapeutic strategy.

Cancer seriously affects human health and is the second leading cause of death in the world. There were 19.3 million new cancer cases and 10.0 million deaths worldwide in 2020. It is estimated that cancer incidence cases will rise to 28.4 million in 2040 [3]. The main cancer treatment modalities include surgery, radiotherapy, chemotherapy, targeted therapy, and immunotherapy [4]. However, poor selectivity, unwanted adverse drug reaction (ADR) and drug resistance of antineoplastic drugs limit their application. In addition, cancer is a gene-driven disease characterized by the diversity of gene mutations between and within individuals [5,6]. Identification of genetic drivers is crucial for advancing cancer therapeutics. In this regard, siRNA therapy has attracted more and more attention due to its great potential in sequence-specific gene regulation. Compared with other therapeutic modalities, siRNA has become a promising platform in the field of cancer therapy due to its inherent advantages. Firstly, siRNA expands the range of therapeutic targets. To date, although 700 oncogene targets have been identified, most of them are “undruggable” [7]. These “undruggable” targets often have undefined structures or lack ligand-binding pockets that are difficult or impossible to target by traditional small compound molecules [8]. RNAi treats diseases at the transcriptional level, which is a form of post-transcriptional gene silencing technology. Since siRNA has the potential to silence any therapeutic gene, and will vastly expand the proportion of therapeutic targets in the human genome, making “undruggable” targets druggable. Secondly, the essence of siRNA drug development is to design the correct nucleotide sequence of the target mRNA, to target any gene you are interested in, making the treatment more precise and personalized. Thirdly, the time required for siRNA drug development is relatively short compared with conventional drugs such as small molecules and antibodies. The development of small molecule drugs or monoclonal antibodies costs a huge amount of money and time, and the target protein is prone to drug resistance once the protein configuration changes, which makes the development of next-generation drugs more difficult. Lastly, siRNA-based therapeutics is safer than cell therapy or gene editing technology because it targets mRNA rather than permanently modifying DNA. In addition, it has been reported that long-term gene silencing efficiency can be achieved by administering siRNA drugs at intervals of six months or even longer, which will offer treatment compliance [9]. Consequently, siRNA-based therapy is a unique and potent way to regulate gene expression by specifically triggering the degradation of mRNAs, and therefore provides an effective therapeutic prospect for cancer patients.

So far, four siRNA drugs have been approved by the Food and Drug Administration (FDA) and European Medicines Agency (EMA) (Table 1). Unfortunately, none of them are used for cancer treatment. Despite their great potential in cancer therapy, siRNA drugs still face some obstacles in clinical application, such as the targeted and effective delivery of siRNAs to extrahepatic tumors and the cytosol of cancer cells. In addition, due to the biological complexity of cancer, combination strategies are usually required to control tumor growth [10].

Numerous outstanding reviews have elaborated the common delivery systems used in siRNA drug development [11,12,13,14]. In this review we focus on recent advances in delivery strategies of siRNA drugs in cancer treatment, including lipid-based nanoparticles (NPs), polymer-based NPs, siRNA-ligand conjugates and exosomes. These nanocarriers are expected to selectively deliver siRNA to cancer cells or immune cells to achieve tumor targeted therapy or tumor immunotherapy. In addition, we also summarize the related therapeutic targets and combined treatment strategies of siRNA drugs according to the characteristics of tumor biology.

## 2. Development and Challenges of siRNA-Based Therapeutics

RNA silencing technology was first proposed in 1998 [15]. In 2001, researchers successfully silenced related mRNAs in mammals by siRNA [16]. In 2003, pharmaceutical companies began distributing RNA-silencing drugs. Currently, Patisiran^®^, Givosiran^®^, Lumasiran^®^ and Inclisiran^®^ have been approved for the treatment of hereditary transthyretin-mediated amyloidosis (hATTR), acute hepatic porpyria (AHP), primary hyperoxaluria type 1 (PH1) and hyperlipidemia, respectively. Although the discovery of this new technology has achieved clinical success in just 20 years, the development of siRNA drugs has not been smooth sailing. This is largely due to the limited pharmacokinetics (PK) behavior of siRNA in systemic application.

**Figure 1:**
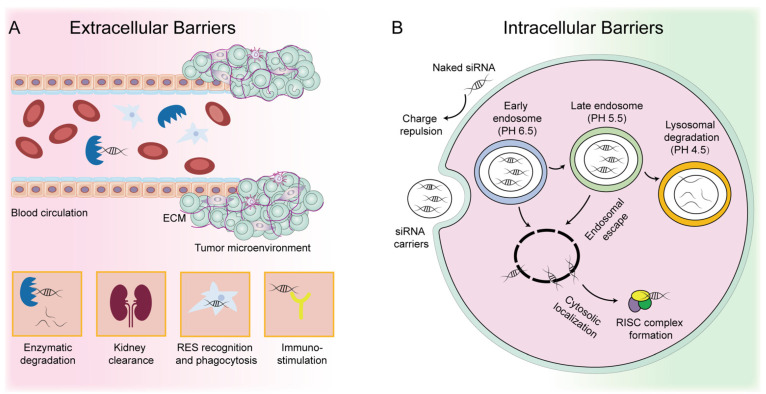
Extracellular and intracellular barriers of siRNA-based cancer therapeutics. (**A**) Extracellular barriers include enzymatic degradation, renal clearance, RES recognition and phagocytosis, activation of immune system and the complex tumor microenvironment. (**B**) Intracellular barriers include endosomal trapping and lysosomal degradation in cancer cells.

siRNA is a typically hydrophilic polyanion with a size of about 13 kDa, which does not pass easily through the plasma membrane. Systemically injected siRNA drugs first encounter extracellular barriers, including enzymatic degradation by endonuclease and RNase, clearance by the kidney, recognition and phagocytosis by the reticuloendothelial system (RES), repulsion by the negatively charged cell membrane, and activation of the immune system (Figure 1A) [17,18]. siRNA faces the complicated tumor microenvironment (TME) and must pass through the dense extracellular matrix (ECM) to enter cancer cells. Even after entering the cells, siRNA encounters intracellular barriers, such as the endosomal trap and lysosomal degradation (Figure 1B). This series of complex extracellular and intracellular barriers has brought great challenges to siRNA therapeutics, leading to the failure of most trials. Thus, the major difficulty and challenge of siRNA application in vivo lies in the successful delivery of siRNA in targeted tissues and cells, as well as its release in the cytoplasm. To overcome this, a great deal of vehicles has been developed for siRNA delivery, including virus and non-virus-based platforms. Although viral vectors have proven to be highly effective in delivering siRNA, the potential safety issues of viral vectors make them less attractive than non-viral vectors.

siRNA drugs also face other challenges and limitations. For example, siRNA is less effective for proteins with a longer half-life because siRNA degrades mRNA rather than proteins that have been synthesized. Although siRNA has the ability to significantly silence target genes, this silencing ability is temporary because siRNA is exogenous and does not occur on its own. With the growth and division of cells, the inhibitory effect of siRNA is weakened. Therefore, repeated administration is required during siRNA treatment. In addition, correct sequence design is an important factor to improve siRNA therapy. For instance, the sequence of the antisense strand (the guide strand that activates RISC binding to the target mRNA) determines the effectiveness of siRNA therapeutics.

To date, all the approved siRNA drugs have focused on hepatic diseases, and none of them are used in cancer treatment. Free siRNA and its carriers can be trapped by the liver due to its fenestrated endothelium and highly perfused property [19,20,21]. In addition, overexpression of certain receptors in hepatocytes, such as asialoglycoprotein receptor (ASGPR), can facilitate the uptake [22]. Hence, the development of effective extrahepatic delivery technologies remains a major goal for siRNA-based cancer therapeutics.

## 3. Strategies to Improve siRNA Delivery in Cancer Therapy

**Figure 2:**
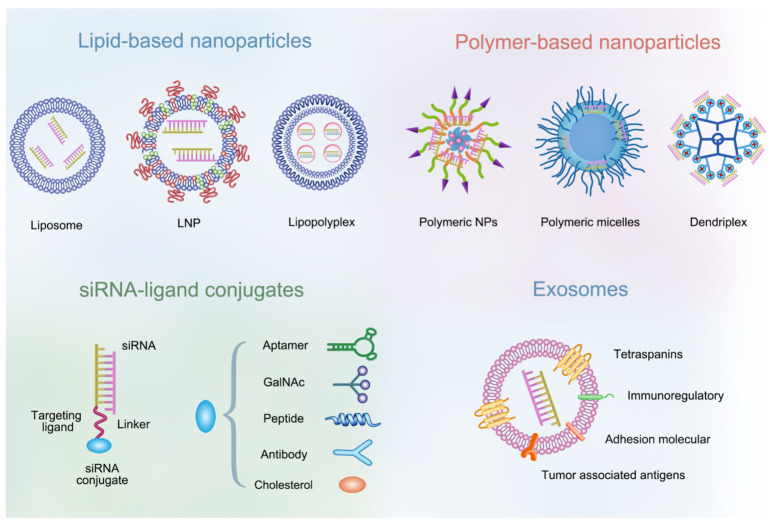
Schematic diagram of non-viral strategies to improve siRNA delivery. The most commonly used delivery systems include lipid-based nanoparticles (liposome, LNP and lipopolyplex), polymer-based nanoparticles (polymeric nanoparticles, polymeric micelles and dendritic polymers), siRNA-ligand conjugates and exosomes.

Chemical modification and nanoparticle (NP)-based delivery systems (e.g., lipid-based NPs, polymer-based NPs, siRNA-ligand conjugates, and exosomes) can optimize the PK characteristics of siRNA, protect siRNA from degradation by nuclease, and prevent it from renal clearance. The siRNA delivery systems most relevant to clinical application are discussed below (Figure 2). These strategies have improved the bioavailability of siRNA and brought siRNA-based drugs into clinical trials [23]. Currently, a variety of siRNA cancer therapeutics based on different delivery strategies are in the early clinical trial stage (Table 2), of which only three have completed Phase I/II clinical studies. ALN-VSP02, a lipid nanoparticle-based formulation that double-targets vascular endothelial growth factor (VEGF) and kinesin spindle protein (KSP), has completed a Phase I clinical trial (NCT00882180). Patients tolerated the twice-weekly dose of ALN-VSP02 well. More importantly, an endometrial cancer patient with liver metastases showed a complete response to ALN-VSP02, which is rare in a phase I clinical trial. However, given the different treatment levels of enrolling patients (some of them have received anti-VEGF therapy) and the heterogeneity of tumors, correlations between the VEGF/KSP inhibition and clinical outcomes is hard to determine [24]. Subsequent clinical trials of ALN-VSP may enroll patients with specific types of tumors who have previously been treated less, but there is no information about the initiation of a phase II clinical study of ALN-VSP on the clinical trial website. TKM-080301, also a lipid nanoparticle formulation delivering the polo-like kinase 1 (PLK1) siRNA, has completed phase II clinical studies in advanced solid tumors. However, it will not be further explored as an anti-tumor monotherapy because of its limited tumor-suppressing effect [25]. Atu027, a liposomal formulation containing siRNA against protein kinase N3 (PKN3) with anti-metastatic activity by inhibiting the expression of PKN3 in the vascular endothelium [26], has completed phase I and II clinical trials (NCT00938574, NCT01808638). In a 28-day phase I clinical trial, Atu027 was well tolerated, and 41% patients (14/34) had stable disease at the end of treatment [27]. In the subsequent phase II trial, Atu027 was combined with gemcitabine for the treatment of metastatic pancreatic cancer [28]. The combination of gemcitabine and Atu027 (twice a week) has shown the efficacy treatment benefit in patients with metastatic tumors.

### 3.1. Chemical Modification

Chemical modification is one of the most effective methods to improve the efficiency of siRNA drug delivery. The naked or unmodified siRNA is readily degraded by nucleases in the systemic circulation. In addition, naked siRNA can be considered an exogenous substance by Toll-like receptor (TLR) and Retinoic Acid Inducible Gene-I-like Receptors (RLRs) to induce immunogenicity [29,30]. Chemical modification is expected to solve the problems of siRNA stability and immunogenicity. The common strategies for chemical modification include: (1) modification of the nucleobase; (2) modification of the nucleic acid backbone (phosphonate), and (3) modification of the ribose sugar moiety. As a result, the modification can significantly improve the pharmacokinetics (PK), pharmacodynamics (PD) and biological distribution of siRNA. For example, methyl modification of the 5-position of pyrimidines is commonly used to enhance the stability of siRNA and increase the affinity for target nucleotide [31]. Phosphodiester bonds are the chemical bonds that nuclease acts on, so phosphorothioate (PS) modification can resist nuclease-mediated degradation [32]. The readily hydrolytic 2′OH on pentose phosphate can be replaced by 2′-fluoro (2′F), 2′-O-methyl (2′O-Me) or 2′-O-methoxyethly (2′-O-MOE), which may significantly enhance the stability of siRNA against serum nucleases and abrogate immune stimulation caused by TLR [33,34,35].

### 3.2. NP-Based Delivery Systems

Through the chemical modification strategy, some difficulty, such as stability against serum nuclease or avoidance of immune recognition, have been greatly optimized. However, there are still some other difficulties to be solved, such as crossing the physiological barrier, improving tumor targeting, escaping the endosome, and entering the cytoplasm. Owing to the intrinsic advantages of NP-based delivery systems, such as low toxicity, low immunogenicity, biocompatibility, high encapsulation, controlled release, easy modification and targeting property based on the enhanced permeability and retention (EPR) effect, nanotechnology has made enormous contributions to the delivery of siRNA over the past several decades [36].

One of the greatest advantages of an NP-based delivery system is that it can help siRNA cross physiological barriers. To deliver siRNA to target cells effectively in vivo, NPs should not interact with blood and extracellular tissue. Apart from that, an efficient NP delivery system should also avoid excretion and improve systemic circulation time. The aggregation of NPs in the blood stream, adhesion to proteins, or interaction with other oppositely charged cell membranes are all related to their physico-chemical properties. The rational optimization of these NP carriers by changing size, structure and surface chemistry could improve the encapsulation efficiency and in vivo stability of siRNA, and prevent the clearance from the mononuclear phagocytic system (MPS) and kidney [37,38]. Common optimization strategies include the following. (1) Modulating the size of NPs. NPs larger than 200 nm are easily filtered out by the MPS and NPs smaller than 10 nm are prone to renal clearance. By controlling the particle size, NPs can be prevented from being filtered by MPS and kidneys, thus enhancing the systemic circulation of siRNA. (2) Surface modification. Positively charged NPs usually bind to negatively charged serum proteins in the blood circulation, making the NPs ineffective. More importantly, the body recognizes hydrophobic NPs as foreign substances, so they are rapidly taken up by the MPS. Coating of hydrophilic polymers such as polyethylene glycol (PEG) or N-(2-hydroxypropyl)methacrylamide (HPMA) can create a cloud of chains at the particle surface, thus repelling plasma protein, avoiding MPS uptake and prolonging blood circulation time [39]. In addition, NPs can improve the PK and PD of siRNA drugs, including more favorable biodistribution. It is well known that the liver is the main metabolic organ, and most drug delivery systems are mainly taken up by the liver, so most siRNA drug are currently used in the treatment of liver diseases. Efficient delivery of NPs to extrahepatic organs remains a challenge. In the field of cancer nanotherapeutics, it is generally believed that NPs can accumulate in the tumor site through the EPR effect owing to the leaky tumor vasculature and poor lymphatic drainage [40]. However, studies have showed that only about 0.7% of the administered NPs reach the tumor site through the EPR effect [41]. Active targeting of NPs by surface modification can not only reduce the accumulation of siRNA in non-target tissues, thereby avoiding unwanted toxic side effects, but also enables effective gene silencing at low doses. For instance, the decoration of tumor specific targeting ligands such as arginine-glycine-aspartic acid (RGD) peptide [42], folic acid (FA) [43], hyaluronic acid (HA) [44] and transferrin (Tf) [45], can help NPs deliver siRNA to desired organs by specifically recognizing corresponding receptors, and promote the endocytosis of NPs through receptor-mediated endocytosis. Remarkably, for most NPs, penetration deep into cancer cell is difficult after accumulation in tumors, resulting in a less satisfactory therapeutic effect. Smaller size (~20 nm) and cationic-charged NPs may facilitate tumor penetration [46,47]. However, smaller size NPs usually show poor tumor retention. Recently, a series of size-switchable strategies have provided a solution to balance the NP size problem between tumor retention and penetration [48,49].

It is worth noting that most NPs enter cells through the endocytosis process [50], most of them are trapped in the endosomes, and only under 2% of them enter the cytoplasm [51,52], which limits the therapeutic effect. Thus, a nanocarrier that is capable of disrupting the endosomal membrane is vital to the efficient endosomal escape and gene silencing effect of siRNA. The common method is to use “proton sponge effect” to promote the escape of siRNA from the endosome [53]. A typical example is polyethyleneimine (PEI), which increases the osmotic pressure of endosomes by absorbing chloride ions and H_2_O, resulting in the destruction of endosomes and the release of siRNA. However, it must be noted that PEI has strong cytotoxicity, which increases with the increase of its positive charge density [54]. Similarly, the introduction of pH-sensitive bond in NPs can promote its rapid disassembly in the acidic cavity of endosome, resulting in efficient endosomal escape [55,56]. Another endosomal escape approach is to modify NPs with a cytosol-penetrating antibody or polypeptides, which are used to form membrane pores to facilitate cargo release [57,58]. Alternatively, ionizable phospholipids, which disrupt endosomal membrane structure (hexagonal transformation) by forming a cone shape at low pH environments, can also be used to enhance endosomal escape [59]. In addition, it has been reported that light stimulation can also promote the efficient endosomal escape of siRNA by generating reactive oxygen species (ROS) to destroy the endosomal membrane [60,61].

#### 3.2.1. Lipid-Based NPs

Lipid-based NPs have been extensively used in drug delivery, including cationic liposomes, lipid nanoparticles (LNPs) and lipopolyplex (LPR). Among them, cationic liposomes are the most widely used carrier for siRNA delivery. Cationic liposomes are complexed with the negatively charged siRNA to form lipoplexes with high siRNA transfection ability [62]. However, cationic liposomes are not suited for the systemic delivery of siRNA because their positive charge causes adverse effects during intravenous administration. PEG modification is usually used to improve systemic delivery of liposomes. Although PEG modification can reduce the toxicity of liposomes and prolong their blood circulation, it also limits the cellular uptake of liposomes and the endosomal escape of siRNA [63]. In addition, PEGylation can cause rapid NPs clearance and a potential adverse reaction by activating the immune system [64]. According to the characteristics of the tumor microenvironment (TME) such as low pH and high glutathione (GSH), pH-responsive [65] and redox-responsive and cleavable PEGylated liposomes [66] have been designed to overcome the limitations of PEG modification. The PEG coating shed from the liposome surface improved the transfection ability of liposomes. Apart from that, as discussed above, surface modification of NPs can improve their active targeting in tumor sites. Liposome can be easily modified with various ligands. As an example, EphA10 is more highly expressed in breast cancer cells than normal cells and is a unique breast cancer marker [67]. Surface modification of liposomes with EphA10 antibody enhanced their cellular uptake in breast cancer, enabling active targetability [68]. In addition, to enhance the endosomal escape of siRNA, some helper lipids (e.g., DOPE) were added to the cationic liposome.

**Figure 3:**
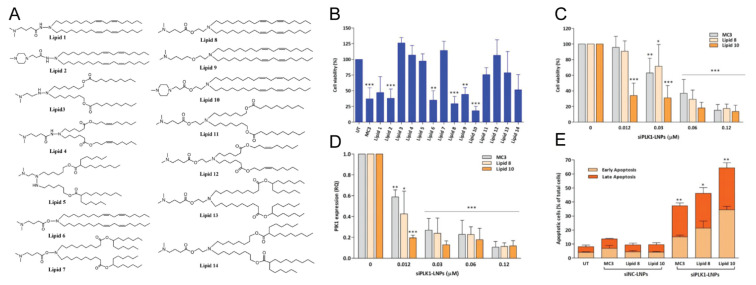
(**A**) Chemical structures of the synthesized new lipids. (**B**) The effect of LNPs-siPLK1 on cell viability. (**C**) The effect of MC3, lipid-8-or lipid-10-based LNPs-siPLK1 on cell viability. (**D**) PLK1 mRNA levels after MC3, lipid-8- or lipid-10-based LNPs-siPLK1 treatment. (**E**) Cell apoptosis analysis after MC3, lipid-8- or lipid-10-based LNPs-siPLK1 treatment. * *p* < 0.05; ** *p* < 0.001; *** *p* < 0.0001. Reprinted with permission from [78]. Copyright 2020 Wiley-VCH.

To date, LNPs have become the most advanced and promising nanomaterials in the field of gene delivery due to their high gene delivery efficiency [23]. Classic LNP formulation consists of cationic or ionizable lipids, cholesterol, saturated phosphatidylcholine (e.g., 1, 2-distearoyl-sn-glycero-3-phosphocholine (DSPC)), and PEG lipids [69]. These components are optimized to efficiently deliver siRNA to targeted cells and achieve gene silencing. Studies have shown that ionizable lipids are less toxic than cationic lipids because the charge of ionizable lipids is dependent on the pH of environment. In order to reduce toxicity and improve delivery efficiency, the acid-dissociation constants (pKa) of ionizable lipids should be lower than 7 to encapsulate siRNA and maintain a neutral surface during the circulation, but should be high enough to become protonated in endosomes [70]. One study has shown that gene silencing efficiency was highest when the pKa value of the lipid was 6.2–6.5. For example, DLin-MC3-DMA (pKa 6.44) exhibited a 10-fold higher efficiency than DLin-KC2-DMA (pKa 6.7) [71]. Onpattro^®^ (patisirna) is the first siRNA drug, approved by the FDA in 2018, for the treatment of hereditary amyloidogenic transthyretin (ATTRv) amyloidosis, which is caused by mutation of the transthyretin (TTR) gene and misfolded aggregates of TTR protein in liver [72,73]. Onpattro^®^ mediated delivery of TTR siRNA by LNPs (formulated from DLin-MC3-DMA) can reduce the average maximum serum TTR by 87.8% for over 18 months. LNP-siRNA mainly accumulates in the liver through the apolipoprotein E (ApoE)-low density lipoprotein receptor (LDLR) pathway after systemic administration, so the majority of LNP technology is used for hepatic gene silencing [74]. This is the LNP’s strength, but it is also a limitation. The development of an extrahepatic delivery system has always been a challenge for the widespread application of LNP technology in cancer therapy because it is not enough to achieve tumor targeting merely relying on the EPR effect. It is also unclear whether the EPR effect occurs in human solid tumors. In recent years, some researchers have attempted to target extrahepatic organs by regulating the composition of LNPs or modifying LNPs [75]. For example, the addition of persistent PEG-lipids (PEG-DSG) could prolong the circulation time of LNPs, but limit the efficiency of gene transfection [76]. To obtain the equal therapeutic effect, the dose of siRNA needed to be increased, which also enhanced the adverse effects of LNPs [77]. Using another strategy, Ramishetti’s group developed and synthesized a series of ionizable amino lipids based on a linker backbone (hydrazine, hydroxylamine and ethanolamine) to improve the delivery of siRNA beyond the liver (Figure 3) [78]. They encapsulated PLK1 siRNA in LNPs to assess their gene silencing efficiency in myeloma suspension cells. Compared with DLin-MC3-DMA (a gold standard ionizable lipid clinically approved for siRNA delivery), lipid8 and lipid10 further inhibited cell viability, reduced PLK1 mRNA expression and induced cell apoptosis. Importantly, these novel lipids did not induce an increase in liver enzymes (SGPT, SGOT) or activate the immune system. Recently, Liu shuai’s group synthesized a series of novel multi-tailed ionizable phospholipids (iPhos) to form multi-component lipid nanoparticles (iPLNPs) for organ-selective nuclei acid delivery [59]. Compared with DOPE and DSPC, iPhos exhibited the characteristics of strong endosomal escape properties, due to the chemical structures of phospholipids. DOPE and DSPC consist of one irreversible zwitterion head and two hydrophobic tails, while iPhos possesses a PH-switchable small zwitterion head and three hydrophobic alkyl tails. A small zwitterion head combined with three hydrophobic tails would more easily form a cone than one with two tails, causing easier membrane phase transition and endosomal escape. Importantly, the length of the iPhos chain determines organ selectivity in vivo. For example, the formulas 9A1P9-5A2-SC8 and 9A1P9-DDAB can preferentially deliver the target nucleic acid to the liver and lung, respectively. Alternatively, extrahepatic targeting can also be achieved by modifying the surface of LNPs with ligands or antibodies [79].

Recently, the COVID-19 pandemic has seriously affected the lives and health of people around the world. Given that LNP is a clinically proven delivery technology, ionizable LNP (iLNP) has been expanded in COVID-19 vaccines against SARS-CoV-2. For example, Moderna and Pfizer/BioNTech use LNPs to encapsulate mRNA encoding the SARS-CoV-2 antigen, resulting in ~95% protection efficacy [80,81]. LNP-based mRNA vaccines can take substantial advantages of rapid development, easy industrialization, and flexibility against new variants. Unlike siRNA, which down-regulates the target protein, mRNA vaccines up-regulate the target protein. Notably, like LNP-siRNA, LNP-mRNA vaccines do not have the potential risk of genome integration. Although LNPs for mRNA delivery are similar in composition to LNPs for siRNA delivery, both of which are composed of phospholipids, cholesterol, PEG-lipid and ionizable lipids, the mRNA vaccines on the market (BNT162B2, mRNA-1273) optimize ionizable lipids. SM-102 (Moderna, mRNA-1273) and ALC-0315 (Pfizer/BioNTech, BNT162B2) have similar structures. For example, the ester linkages in the lipid tail were introduced to promote their elimination via hydrolysis, tertiary amino alcohols in the headgroup (aminoethanol headgroup/SM102, aminobutanol headgroup/ALC-0315) were added to increase hydrogen-bonding interactions with mRNA, and the more branched alkyl tail was added to form cone-structure [82,83]. ALC-0315 (Pfizer/BioNTech), the ionizable lipid part of BNT162B2, was designed for mRNA delivery to prevent COVID-19. Researchers used an LNP/ALC-0315 delivery system to deliver siRNA and found that its siRNA silencing efficiency was 2-fold (FVII) and 10-fold (ADAMTS13) higher than that of LNP/DLin-MC3-DMA (an LNP formulation specifically designed for siRNA delivery). This may be because, unlike DLin-MC3-DMA (two tails), ALC-0315 has two branched tails (four tails), forming a more pronounced cone-shaped structure that facilitates endosomal escape, as described previously. However, it should be noted that a high dose (5 mg/kg siRNA) of LNP/ALC-0315 has hepatotoxicity with elevated levels of ALT and bile acid, but LNP/DLin-MC3-DMA has not [84]. Although the composition change, modification or functionalization of LNPs can facilitate the uptake, release of siRNA and the delivery of siRNA to other organ sites, it increases its complexity and toxicity. Indeed, there have been clinical trials of LNP formulation delivering siRNA for cancer treatment (e.g., TKM-080301, DCR-MYC, NBF-006), but some serious adverse events have been observed in subjects [25]. Therefore, researchers must consider optimizing the composition and proportion of LNP components to reduce toxicity without affecting siRNA efficiency, which will be a main research direction in the future.

#### 3.2.2. Polymer-Based NPs

**Figure 4:**
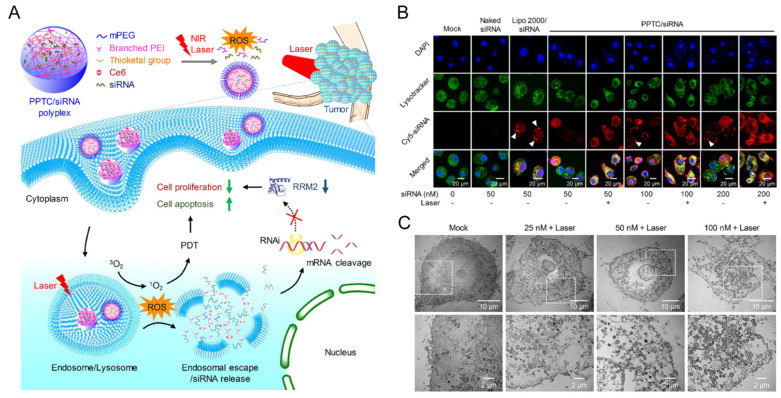
(**A**) Illustration of the ROS-activatable PPTC/siRNA polyplex co-delivering ce6 and siRNA. (**B**) CLSM images of HepG2-Luc cells after coincubation with different concentration of PPTC/siRNA for 4 h with or without laser irradiation (0.1 W/cm^2^, 2 min). (**C**) TEM image analysis after HepG2-Luc cells were treated with PPTC/siRNA polyplex and irradiated with 660 nm laser (0.1 W/cm^2^, 2 min). Reprinted with permission from [97]. Copyright 2020 American Chemical Society.

Polymers are another ideal gene delivery platform [85]. Cationic polymers compress nucleic acids into multi-stranded bodies through electrostatic interaction, which enhances cellular uptake and lysosomal escape due to positively charged groups [86]. In this respect, polyethyleneimine (PEI) is the most commonly used cationic polymers for siRNA delivery, with linear and branch types [87]. However, PEI is highly cytotoxic due to its strong positive charge. Therefore, the surface charge should be considered carefully when designing a PEI-based carrier; for instance, by shielding PEI with compounds such as PEG. On one hand, the PEGylation of PEI can significantly reduce the toxicity caused by PEI and improve the solubility of polymer NPs. However, on the other hand, the PEG layer hinders electrostatic interaction between the cationic polymer and negative siRNA, which causes more polymer usage and low transfection efficiency. To improve transfection efficiency and reduce toxicity, low molecular weight PEI is usually conjugated with other ligands such as cyclodextrins (CD) [88], chitosan [53] and hyaluronic acid (HA) [89]. In addition, other synthetic polymers, such as cationic dendrimers with multiple branches, are also used for siRNA delivery because of their clearly-defined molecular structures and functional groups [90]. Recently, a variety of dendrimers have been well-studied for siRNA delivery, such as poly(propylenimine) (PPI), polylysine (PLL) and poly(amidoamine) (PAMAM). PLL, a peptide-based dendrimer, has better biocompatibility and biodegradability than PEI. A study has shown that conjugation of PEI with PLL could enhance the transfection efficiency and reduce the cytotoxicity of PEI [91]. Patil et al. designed a poly(amido amine)-poly(ethylene glycol)-poly-l-lysine (PAMAM-PEG-PLL) triblock polymer, which was proved to be an efficient gene carrier for silencing Bcl-2 expression [92]. The complexity and diversity of cancer promote the development of versatile nanomaterials. One example is that smart responsive polymer-based NPs can be designed based on the unique microenvironmental characteristics of tumors different from normal tissues, such as enzyme/pH/GSH/ROS-sensitive polymers. Apart from the intrinsic stimuli response, smart polymers sensitive to extrinsic stimuli such as ultrasound, temperature, and light/laser irradiation, have been extensively studied in recent years. In addition, to achieve targeted delivery, polymers can be conjugated with diverse ligands. Furthermore, a hydrophobic segment could be added in cationic water-soluble polymers to form amphiphilic polymers multi-functionality. Some of the most common hydrophobic moieties are poly (lactide-co-glycolide) (PLGA) [93], PLA [94], PCL [95], and photosensitizers (e.g., porphyrin) [96]. These amphiphilic polymers can self-assemble into core-shell structures in aqueous solution. The hydrophobic inner core could be used to deliver hydrophobic drugs (e.g., DOX, PTX etc.) or realize photothermal therapy (PTT)/photodynamic therapy (PDT), while the hydrophilic shell could be used to maintain water solubility, and the cationic segment could be used to absorb siRNA. In a study reported by Zhang Mengjie et al., a ROS-activatable, siRNA-engineered polyplex (PPTC/siRNA) composed of a PEGylated cationic polymer (PEI), ROS-cleavable linker (thioketal), Ce6 (photosensitizer), and RRM2-siRNA, was constructed to promote siRNA endosomal escape, enhance cell apoptosis, and inhibit cell proliferation (Figure 4) [97]. PDT is known to generate ROS, which not only promotes cell apoptosis but also triggers the cleavage of the ROS-sensitive linker in this formulation to enhance endosomal escape. This multifunctional system successfully reduced the expression of RRM2 and enhanced the antitumor effect of RNAi and PDT. Hence, the development of multifunctional polymers may not only solve several obstacles of siRNA therapeutics, but also realize multimodal integration, which will have a significant impact on cancer therapy. However, combining all components into one platform for multi-functionalization also adds to the complexity of delivery systems, and how each component performs its function, and whether it interacts with others, should be clearly studied. In particular, in-depth information on the distribution, circulation, toxicity of each part is necessary for successful clinical translation.

#### 3.2.3. siRNA-Ligand Conjugates

The conjugation of siRNA with ligands can enhance their cellular uptake and reduce toxicity, which is a promising delivery strategy. Gene silencing requires the 5′ end of the antisense strand, so conjugation is usually performed on the sense strand’s 5′ end or the antisense strand’s 3′ end. Ligands include cholesterol, small molecules, aptamers, peptides and antibodies [98,99]. Various ligands have different functions to achieve specific receptor targeting, and a ligand that can specifically bind a certain receptor expressed by cancer cells can be chosen for conjugation. For example, prostate-specific membrane antigen (PSMA) is overexpressed on the surface of prostate cancer cells, and can be used to specifically target prostate cancer [100]. In addition, N-acetylgalactosamine (GalNAc) siRNA conjugates have achieved great success in liver-targeted delivery and clinical translation. GalNAC-siRNA has high tissue-targeting specificity, small size (compared with NPs), high therapeutic index and minimal adverse effects [101]. The asialoglycoprotein receptor (ASGPR) is highly expressed on hepatocytes that specifically bind to its ligand (GalNAc) and cause endocytosis, thus achieving liver-targeted drug delivery [102]. Givosiran^®^ (Givlaari), a GalNAC-siRNA based on enhanced stabilization modification chemistry (ESC), were developed by Alnylam Pharmaceuticals for the acute hepatic porphyria (AHP) treatment [103]. Clinical studies have shown that GalNAC-siRNA is more suitable for subcutaneous administration than intravenous administration and can be administered once a month. In addition, a second GalNAc-conjugated siRNA (Lumasiran^®^, Oxlumo) [104] and a third GalNAc-conjugated siRNA (Inclisirna^®^, Leqvio) [105] have also been approved for the treatment of primary hyperoxaluria type 1 (PH1) and hyperlipidemia, respectively. However, the drawback of siRNA-ligand conjugates is the lack of components that promote the endosomal escape of siRNA. Nevertheless, enough siRNA accumulation in endosome can compensate for the lack of endosomal release. For example, efficient cellular uptake, fast receptor recycling and chemical structure stabilized siRNA make GalNAc-conjugated siRNA sufficiently accumulated in cytoplasm, although <1% of GalNAc-siRNA is released from endosome [101,106]. Thus, the finding of high numbers of rapidly converting and recyclable receptors to increase endosomal accumulation is the key challenge for endosomal escape of siRNA-ligand conjugates. More importantly, a ligand-mediated delivery system is safer than other delivery platforms (such as LNPs) due to its easy sample formulation and reduced complex materials. For example, the incidence of adverse events related to GalNAc-siRNA drugs was lower than that of LNPs in clinical trials [107,108].

#### 3.2.4. Exosomes

**Figure 5:**
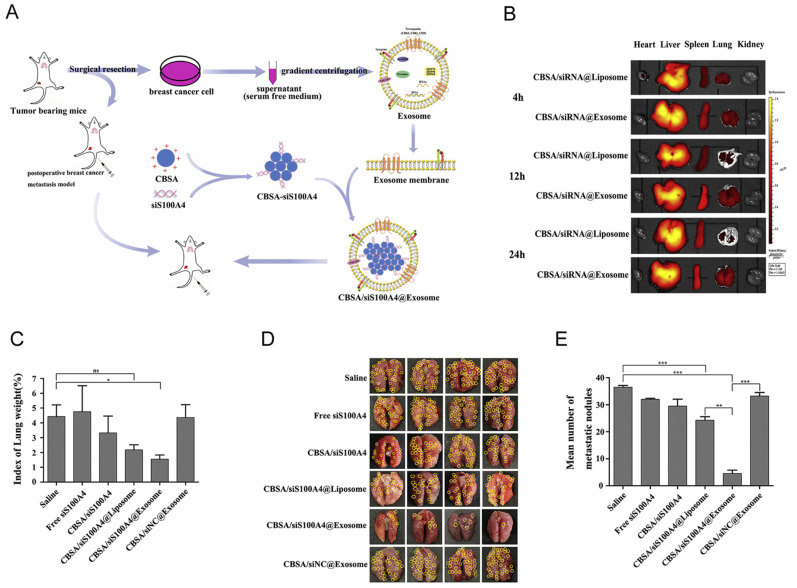
(**A**) Illustration of the CBSA/siS100A4@exosome to suppress postoperative breast cancer lung metastasis. (**B**) EX vivo images of major organs from mice treatment with CBSA/siRNA@Liposome and CBSA/siRNA@Exosome at different time points post-injection. (**C**) Weight differences in lung tissues after treatment with saline, free siS100A4, CBSA/siS100A4, CBSA/siS100A4@Liposome, CBSA/siS100A4@Exosome and CBSA/siNC@Exosome. (n = 4, * *p* < 0.05; ns, not significant). (**D**) Lung tissue images of postoperative lung metastases mice after treatment with saline, free siS100A4, CBSA/siS100A4, CBSA/siS100A4@Liposome, CBSA/siS100A4@Exosome and CBSA/siNC@Exosome (metastatic nodules were marked by yellow circles). (**E**) Mean number of lung metastatic nodules of different treatment groups (n = 4, ** *p* < 0.01; *** *p* < 0.001). Reprinted with permission from [115]. Copyright 2019 ELSEVIER B.V.

Recently, exosome or extracellular vesicle (EV)-mediated siRNA delivery systems have attracted tremendous attention, some of which have reached clinical trials [109]. Compared with other synthetic vehicles, exosomes have lower immunogenicity and higher biocompatibility due to their natural origin [110]. Methods of loading siRNA into exosomes include electroporation [111,112], sonication [113], incubation [114], and liposome/cationic complex-EV formation [52,115]. Exosomes are natural nano-sized vesicles (40∼100 nm) released from cells, which have distinct biological characteristic depending on cell types [116]. Exosomes derived from different cells carry specific markers and have homing characteristics, which may increase the enrichment of a drug within the target tissue [117,118]. On this basis, Zhao liuwan’s group isolated exosomes with lung-targeting ability from autologous breast cancer cells for the treatment of lung metastases in triple-negative breast cancer (TNBC) (Figure 5) [115]. They first synthesized cationic bovine serum albumin (CBSA) that can be used to encapsulate metastasis-related therapeutic siRNA (S100A4 siRNA) via electrostatic interaction. Meanwhile, exosome membranes were extracted from exosomes isolated and purified from autologous breast cancer cells. Finally, the CBSA/siRNA complex and exosome membranes were incubated and extruded to fabricate the CBSA/siRNA@Exosome (Figure 5A). This nanoplatform was able to target the lung (Figure 5B), and also significantly inhibited lung metastasis through its lung targeting function and the RNAi effect of S100A4 siRNA (Figure 5C–E). The presence of MHC I/II on the surface of antigen-presenting cell (APC)-derived exosomes enhanced the immune response by the antigen-presenting function [119,120]. This suggests that this type of exosome can not only be used as a vector, but also can be used for enhancing cancer immunotherapy. Cancer-derived exosomes can influence the TME, and integrins on cancer-derived exosomes can determine organotropic metastasis [121]. Therefore, exosomes are superior carriers in the treatment of cancer. Studies have shown that folic acid (FA)-displaying exosomes mediate the cytosolic delivery of siRNA, which avoids endosomal trapping and improves delivery efficiency [122]. A phase I clinical trial has been undertaken to study the therapeutic effect of KRAS G12D siRNA delivered by mesenchymal stromal cell-derived exosomes in pancreatic cancer patients (NCT03608631). However, it is difficult to obtain homogeneous and high-yield exosomes, which are essential for enhanced therapeutic effect and clinical application. Thus, the extraction and purification techniques of exosomes should be improved to promote industrial production.

## 4. Potential Targets for siRNA-Based Cancer Therapeutics

In the past few decades, siRNA-based cancer therapy has made considerable progress. However, among a large number of successful preclinical studies, only a few (about 10) have entered clinical trials. In addition to the elaborate design of nano-delivery systems, the selection of target genes must also be considered, as a better understanding of the behavior of oncogenes can facilitate precise treatment. Since siRNA has the ability to reduce the expression of target genes, studies have focused on using siRNA to down-regulate cancer-associated genes, including undruggable genes (e.g., KRAS, MYC) [123]. The potential gene targets for siRNA-based cancer therapy mainly include: (1) genes that promote tumor growth, such as tumor driver genes; (2) genes that supply tumor nutrients, such as angiogenesis genes and metabolism-related genes; (3) genes that help cancer cells develop drug resistance and metastasis, and (4) genes that modulate TME, such as cancer-associated fibroblasts (CAF), immunosuppressive cells, and immune checkpoints.

### 4.1. Genes Promoting Tumor Growth

Cancer driver genes (also called oncogenes) refer to those genes that cause tumor growth by mutation or amplification. The activation of oncogenes and the loss of function of tumor suppressor genes leads to uncontrolled cell growth. As mentioned above, about 700 oncogenes have been identified, which are important targets for cancer treatment [7]. However, only a few of these genes have been extensively studied for cancer treatment, including KARS [124], MYC [125], EGFR [126], FGFR [127], BRAF [128], PLK1 [129], EphA2 [130]. KRAS is the most mutated oncogene in cancer [124], with mutations in non-small cell lung cancers (NSCLC) (20-25%) [131], colorectal cancers (30–50%) [132] and pancreatic ductal adenocarcinoma (95%) [133]. However, due to the lack of an accessible hydrophobic pocket to which the drug can bind, KRAS was once considered an “undruggable” target. The main advantage of siRNA therapy is that it does not need to consider the druggability of the protein, so it can target any cancer driver gene. In addition, targeting genes that promote tumor growth will lead to longer gene silencing by preventing cell proliferation. In a study reported by Sushrut Kamerkar et al., tumor growth in multiple mouse models of pancreatic cancer was suppressed by iExosomes/KRAS^G12D^siRNA [123]. Polo-like kinase 1 (PLK1), a well-known serine/threonine-protein kinase, is considered an oncogene regulating the cell cycle and negatively modulating the function of the p53 gene [129]. Arbutus Biopharma Corporation has developed a PLK1 siRNA drug (TKM-080301) against adrenocortical carcinoma that is consist of SNALP encapsuled with siRNA. In a phase II clinical study (NCT01262235), TKM-080301 demonstrated preliminary anti-tumor efficacy [134]. In addition, the increased expression of genes involved in apoptosis, autophagy and the cell cycle can also promote the occurrence and development of cancer. Di et al. used phenylboronic acid (PBA)-functionalized amine-terminated polyamidoamine (PAMAM) to deliver Bcl-2 siRNA, and inhibited the proliferation of hepatocellular carcinoma cells by down-regulating the apoptosis protein Bcl-2 [135].

### 4.2. Genes Supplying the Tumor Nutrients

In order to meet the needs of oxygen and nutrients for tumor proliferation, the tumor forms new blood vessels with the help of angiogenic factors. For example, VEGF is thought to influence tumor angiogenesis and blood vessels growth, thus supplying nutrients for tumor growth [136]. Wang gangmin et al. synthesized polyethylene glycol-poly(ε-benzyloxycarbonyl-l-lysine) (PEG-SS-PLL) block copolymer as a non-toxic and efficient siRNA nanocarrier for anti-angiogenesis therapy. They demonstrated that this VEGF-siRNA delivery system significantly inhibited the expression of VEGF protein in tumor tissue and inhibited the growth of hepatocellular carcinoma [137]. Another example is targeting glycolysis proteins. Despite the genetic diversity in tumorigenesis, tumor cells exhibit a common set of functional characteristics, including tumor cells preferring glycolysis to oxidative phosphorylation, which is known as the Warburg effect [138]. Recently, scientists have sought to achieve targeted tumor therapy by inhibiting the activities of key enzymes in the tumor glycolysis pathway [139]. Key enzymes in glycolysis, such as hexokinase 2 (HK2), pyruvate kinase M2 (PKM2) and phosphofructokinase (PFK) have become new tumor biomarkers, so targeting these genes can turn off the nutrient and energy sources of tumors. Among these genes, PKM2 is of great significance in effective cancer therapy due to its crucial role in promoting the proliferation and invasion of cancer cells [140]. Dang juanjun et al. synthesized a type of guanidine-rich, spherical helical polypeptide (DPP)-based nanocarrier to deliver PKM2 siRNA, which sensitized photothermal therapy by inhibiting tumor glycolysis [141].

### 4.3. Genes Promoting Tumor Drug Resistance or Metastasis

Chemotherapy is one of the most conventionally used methods for cancer treatment, while multi-drug resistance (MDR) is one of main reasons clinical cancer chemotherapy fails. MDR of tumors can be divided into two types, with ATP-dependent effluent pumps and non-ATP-dependent effluent pumps [142]. ATP-dependent extravasation pumps are mainly characterized by abnormal expression of ATP-binding cassette (ABC) transporters, which included P-glycoprotein (P-gp), multidrug resistance-related proteins (MRP), as well as breast cancer resistant proteins (BCRP) [143]. These ABC proteins mainly rely on membrane-bound efflux pumps of active drugs, and transport substances out of cells by the energy released by hydrolysis of ATP against a concentration gradient, which is closely related to the drug resistance of related doxorubicin (DOX) and platinum chemotherapeutic drugs [144]. Non-ATP-dependent efferent pump MDR does not depend on the energy generated by ATP hydrolysis but directly reduces the ability of anticancer drugs to induce cell death, including DNA damage repair and anti-apoptosis. Zhang meng et al. constructed an siRNA-based vesicle (siRNAsomes) for co-delivery of DOX and P-gp siRNA to treat drug-resistant breast cancer. An siRNA-SS-PNIPAM deblock copolymer was formed via exchange reaction between PNIPAM orthopyridyl disulfide (PNIPAM-SS-Py) and mercapto siRNA (siRNA-SH), which self-assembles into siRNAsome upon heating because PNIPAM is temperature-responsive. SiRNAsomes consist of a hydrophilic siRNA shell, a hydrophobic median layer, and an empty aqueous interior. In this study, siRNAsomes reduced the mRNA level of P-gp by approximately 42% in MCF-7 MDR cells, and showed synergistic anti-tumor therapeutic efficacy in MCF-7/ADR mouse xenograft models [145]. It is worth noting that more than 40% of NSCLC patients have EGFR mutations. Although the 1–3 generations of EGFR-tyrosine kinase inhibitors (TKI) can significantly prolong the survival of patients, drug resistance limits their clinical application. Notably, the activation of bypass signaling pathways such as HER2, IGF1R, AXL and BRAF can render tumor cells resistant to EGFR-TKI therapy [146]. It is possible to obtain benefit by constructing specific siRNAs against EGFR-TKI resistance associated with bypass genes. For example, IGF1R specific siRNA significantly restored the sensitivity to Osimertinib (the third generation EGFR-TKI) in Osimertinib-resistant cells [147].

Tumor metastasis is another important factor in the failure of cancer treatment, and is also responsible for most cancer-related deaths. Cancer metastasis is a multistep cascade process with plasticity at the epigenetic and genetic levels. A typical example is that tumor cells undergo epithelial-mesenchymal transition (EMT) to induce cell migration, invasion, intravasation and extravasation [148]. Recent studies have confirmed that tumor metastasis is associated with excessive activation or dysfunction of certain genes, such as PTPN, LCN2, TGF-b, Twist, NF-κB, Snail and S100A4. For example, Shan Tang et al. reported two amphiphilic polymers (PEI-PDHA and PEG-PDHA) to co-deliver Twist/Snail siRNA and paclitaxel for metastatic breast cancer treatment, resulting in decreased metastatic nodules in the lungs [149].

### 4.4. Genes Modulating the TME

TME is composed of normal stromal cells (fibroblasts, immune cells, endothelial cells and pericytes), extracellular matrix, and a variety of soluble factors (cytokines and growth factors) with biological functions, which closely interacts with tumor development and highly modulates tumor response to cancer treatment [150]. Targeting the TME is a new and promising way to treat cancer.

#### 4.4.1. Targeting Cancer-Associated Fibroblasts

Cancer-associated fibroblasts (CAFs) belong to a class of stromal cells in the TME that can promote the growth, metastasis, and drug resistance of tumor cells through secreting various cytokines, chemokines, as well as extracellular matrix (ECM). Some studies have found that CXCL12 secreted by CAF directly promotes cell migration, survival, and proliferation via the CXCL12/CXCR4 pathway. Down-regulation of CXCL12 may be an effective way to inactivate CAF-induced tumor proliferation. Therefore, a new type of cell-penetrating peptide (nine-arginine, R9)-based self-assembled NPs with surface modification with fibroblast activation protein-α monoclonal antibodies (anti-FAP-α mAb) has been developed for the targeted delivery of CXCL12 siRNA (PNP/siCXCL12/mAb) [151]. The results show that PNP/siCXCL12/mAb specifically delivered siRNA into CAFs by targeting the FAP-α on the cell membrane of CAFs, and inhibited the CXCL12 genes expression in CAFs, thus effectively reshaping the TME associated with CAFs. Thus, migration, invasion, and angiogenesis of tumor cells were potently inhibited, thereby suppressing the metastasis of an orthotopic prostate tumor.

#### 4.4.2. Targeting Immunosuppressive Cells or Immune Checkpoints

Most cancer therapy usually failed with tumor recurrence because patients do not fully respond to commonly used therapies, including chemotherapy, surgery and radiotherapy. Unlike commonly used therapies that directly kill cancer cells, cancer immunotherapy may produce a lasting immune surveillance effect by activating immune cells to avoid cancer recurrence. Most solid tumors have an immunosuppressive TME, which is rich in myeloid-derived suppressor cells (MDSC), tumor-associated macrophages (TAMs) and regulatory T cells (Treg) [152]. The immunosuppressive TME limits the efficacy of immunotherapy. Regulation of immunosuppressive TME and activation of immune effector cells are emerging therapies for cancer. TAMs, the most abundant innate tumor-infiltrating immune cells in tumors, including the M1 classic activation phenotype and M2 alternative phenotype [153]. TAM tends to polarize to the activated M2 phenotype that inhibits the tumor immune microenvironment and promotes tumor growth by producing mediators that remodel the tumor supportive TME. For example, M2-like TAM may inhibit the efficiency of dendritic cells (DCs) and promote the expression of pro-angiogenic growth factors (VEGF, FGF) [154,155]. Thus, targeting M2-like TAMs is an attractive strategy for cancer immunotherapy. Depleting and reprogramming TAMs are main strategies for reversing the immunosuppressive TME triggered by M2-like TAMs. Studies have shown that blocking the CSF1/CSF1R axis can re-educate TAMs. Yuan Qian et al. prepared M2-like TAM dual-targeting nanoparticles (M2NPs) composed of biocompatible fusion peptide-functionalized lipid [156]. M2NPs selectively delivered anti-colony stimulating factor-1 receptor (anti-CSF-1R) siRNA to M2-like TAMs and eliminated 52% of M2-like TAMs, resulting in 87% reduction in melanoma tumor volume and prolonged survival, indicating that an immune memory that inhibits tumor recurrence was established. The programmed cell death protein 1 (PD-1)/programmed cell death ligand 1(PD-L1) axis regulates cancer immunity by modulating T cells activity, and plays an important role in cancer immunotherapy [157]. The overexpression of PD-L1 in the TME of various tumor types makes it a potential target for TME remodeling. Chunhui Li et al. elaborately designed and synthesized a novel pH-responsive hydrophobic core based on a three-block polymer mPEG_45_-P (DPA_50_-co-DMAEMA_56_)-PT_53_ (PDDT), which can efficiently load PD-L1 siRNA and achieve rapid endosomal escape [55]. They conducted immune surveillance in PD-L1 overexpressed colon carcinoma cell line (CT-26) models by enhancing the function of CD8+ and CD4+ T cells by silencing PD-L1, which ultimately led to effective tumor inhibition and longer survival.

## 5. Combined Strategies with Other Therapeutic Modalities

Although siRNA-based therapeutics can significantly reduce the expression of overexpressed cancer-associated genes, treatment using single specific gene inhibition has difficulty in completely eliminating the tumor and may even cause other bypass pathway activation because of multi-gene alterations and heterogeneity of tumor [158]. The integration of multiple treatment modalities will potentially maximize the anti-tumor therapeutic effect and overcome the shortage of individual treatments. Current clinical trials support this view. The clinical response of mono-siRNA therapeutics (Atu027, CALAA-01, TKM-PLK1, DCR-MYC) are either disease stabilization, partial response or disease progression [159]. ALN-VSP02 contains two siRNAs targeting VEGF and KSP, which are associated with cancer angiogenesis and proliferation, respectively. A phase I clinical trial of ALN-VSP02 demonstrated that one patient with liver metastases in endometrial cancer achieved a complete response [24]. In addition, promising clinical responses were also observed in the combination of an siRNA drug (Atu027, siG12D-LODER) and chemotherapy [28,160]. Therefore, the incorporation of siRNA therapeutics in other therapeutic modalities will obtain great advantages over monotherapy in cancer treatment, but only when the combined targets and therapeutic modalities are chosen correctly. In general, the purpose of combination strategy can be divided into the following categories: (1) enhance the therapeutic effect of these individual therapeutic modality without overlapping toxicity; (2) relieve the side effects of one therapeutic modality, and (3) reverse drug resistance.

### 5.1. Combined Gene Therapy

As discussed above, dual-target gene therapy has shown more efficacious therapeutic outcomes and advantages than mono gene therapy. Kuan-Wei Huang’s group encapsuled siRNAs and plasmid DNA targeting two genes in one formulation. They designed a type of tumor-targeting lipid-dendrimer-calcium-phosphate (TT-LDCP) NP to co-deliver immune checkpoint ligand PD-L1 siRNA and immunostimulatory IL-2 encoding plasmid DNA. In this nano-delivery system, the 50% thymine-capped PAMAM dendrimer could stimulate innate immunity and improve gene transfection efficiency, which led to the infiltration and activation of CD8^+^ T cells, and ultimately improved the immunotherapeutic effect of hepatocellular carcinoma (HCC) [161]. This strategy of integrating two different gene drugs for cancer therapy should consider the following issues: (1) one single or two different delivery systems; (2) simultaneous or sequential therapy, and (3) rational arrangement of dosage to achieve an optimal ratio.

### 5.2. SiRNA Therapeutics Combined with Chemotherapy

Chemotherapy is still the most common, mainstream treatment for cancer. Recently, a variety of combinations of siRNA and chemotherapeutic drugs have attracted more attention, mainly enhancing anti-cancer effects by inducing programmed cell death [162] or overcoming drug resistance [163]. Pancreatic cancer is known to be a refractory tumor with a 5-year relative survival rate of 10% [164]. Mutated KRAS is considered to be an oncogene for pancreatic cancer. KRAS used to be a “undruggable” gene due to the lack of a drug-binding surface receptor. This drawback has been overcome by RNAi technology. For instance, KRAS^G12D^ siRNA in combination with chemotherapy (Gemcitabine + nab-paclitaxel) was used to treat advanced pancreatic cancer in a phase 2 clinical study (NCT01676259). In another study, O6-methylguanine-DNA methyltransferase (MGMT), a DNA repair protein, mediated temozolomide (TMZ) resistance by eliminating TMZ-induced DNA lesion. Wang kui et al. developed an iron oxide-based nanoparticle system (NP-siRNA-CTX) to deliver MGMT siRNA. Consequently, NP-siRNA-CTX significantly suppressed MGMT gene expression and prolonged survival in an orthotopic glioblastoma model [165]. Some chemotherapeutic drugs work by promoting apoptosis of tumor cells [166]. In some cases, cancer could induce anti-apoptosis protein expression in response to chemotherapy [167]. Therefore, the combination of anti-apoptotic siRNA and chemotherapeutic drugs can improve the therapeutic effect. For this purpose, Suo aili’s group synthesized a folate-decorated PEGylated triblock copolymer (PAH-b-PDMAPMA-b-PAH). This copolymer selectively co-delivered DOX and Bcl-2 siRNA to MCF-7 cancer cells and responsively released cargo in cells due to pH-responsive hydrazone bonds and a reduction-sensitive disulfide linkage, thus promoting cell apoptosis and enhancing the antitumor effect [168]. As mentioned above, P-gp, encoded by *MDR1*(*ABCB1*), functions as an ATP-dependent efflux pump that is involved in taxane drugs resistance. Zhang jiulong et al. overcame multidrug resistance by using pH-sensitive lipoplexes to co-deliver MDR1 siRNA and doxorubicin [68].

### 5.3. SiRNA Therapeutics Combined with Radiotherapy

Radiotherapy is another common cancer treatment modality, causing tumor cell death by inducing DNA double-strand damage [169]. However, patients can have resistance to radiotherapy due to the activation of hypoxia-induced factor 1-α (HIF-1α) under hypoxic conditions [170]. Inhibition of HIF-1α expression to sensitize radiotherapy has attracted much attention. To overcome hypoxia-induced radiation resistance, Yong yuan et al. have developed a type of Gd-containing polyoxometalates-conjugated chitosan (GdW10@CS) nanosphere to deliver HIF-1α siRNA for simultaneous extrinsic and intrinsic radiosensitization. On one hand, as external radiosensitizer, the GdW10@CS nanosphere generated and accumulated more efficient ROS via X-ray irradiation and elimination of the intracellular GSH. On the other hand, as internal radiosensitizer, the GdW10@CS nanosphere inhibited the repair of damaged DNA by silencing HIF-1α expression [171]. The therapeutic efficacy of siRNA combined with radiotherapy was significantly enhanced in vivo, resulting in the inhibition of tumor growth and relapse.

### 5.4. SiRNA Therapeutics Combined with Photodynamic/Photothermal Therapy

Photodynamic therapy (PDT) is an emerging cancer treatment modality that needs a combination of photosensitizer, molecular oxygen and light of specific wavelength to generate reactive oxygen species (ROS) that induce tumor cells death via apoptosis or necrosis [172]. Depending on the therapeutic purpose, the combination of siRNA therapeutics and PDT can be additive or synergistic. FOXA1 is a key transcription factor in breast cancer that can activate estrogen receptor (ER)-dependent genes and promote the proliferation of breast cancer. Zhao ranran et al. recently developed a combination therapy using multi-functional cationic porphyrin microbubbles (CpMBs) for dual delivery FOXA1 siRNA and photosensitizer (porphyrin) in ER-positive breast cancer, resulting in significantly ER+ breast tumor growth inhibition and recurrence [173]. In addition, more recently studies have shown that PDT can stimulate the immune response in the host. However, PDT-mediated cancer immunotherapy is severely limited by the PD1/PD-L1 immune checkpoint pathway [174]. Combined PDT and immune checkpoint inhibitors may enhance the efficacy of immunotherapy. Wang dangge et al. synthesized a class of acid-activatable versatile micelleplexes (POP-PD-L1) to enhance PDT-mediated cancer immunotherapy by inhibiting the expression of PD-L1 in cancer cells. The results demonstrated that the combination strategy of PDT and PD-L1 siRNA significantly inhibited the tumor growth and metastasis of B16-F10 melanoma xenografts [175]. As described previously, the photosensitizer is highly hydrophobic and can be used as hydrophobic core to deliver water-insoluble compounds. Incorporating photosensitizer as a hydrophobic core in an siRNA polymer delivery system can achieve the photodynamic therapeutic effect while delivering anti-cancer drugs. To this end, our group constructed a new class of photosensitizer (pyropheophorbide a)-based amphiphilic and block dendritic polymer (Polymer) that co-delivers gefitinib and YAP-siRNA to achieve drug/gene/photodynamic cocktail therapy [176]. In both Gef-resistant NSCLC cell line-derived xenograft (CDX) and patient-derived xenograft (PDX) models, Polymer@Gef-YAP-siRNA showed excellent antitumor effects. Apart from PDT, PTT is another extensively studied treatment modality that inhibits tumor growth by laser irradiating the tumor site to produce hyperthermia [177]. However, PTT requires maintaining the temperature of tumor site at over 50 °C, which may potentially damage normal tissues and induce tumor metastasis. Recently, PTT under mild temperature has received more attention. Traditional PTT promotes tumor cells death mainly through high temperature-induced cell necrosis, whereas low temperature PTT triggers tumor cells death via a programmed cell death mechanism such as apoptosis. Recent studies have shown that heat shock protein (HSP70) is associated with the PTT-induced cell death pathway and promotes cell thermoresistance [178]. Fei Ding et al. demonstrated that HSP70 siRNA combined with low-temperature (42~45 °C) PTT could achieve an effective antitumor effect by sensitizing PTT [179]. They fabricated a polydopamine (PDA)-coated nucleic acid nanogel (PEG-PDA-Nanogel) which could realize low-temperature PTT mediated by HSP70 siRNA. This nanogel utilized PDA and HSP70 siRNA for the generation of insufficient hyperthermia and downregulation of HSP70, respectively, resulting in cell apoptosis rather than necrosis.

Overall, siRNA will bring benefits to other therapeutic modalities and achieve win-win results if the delivery problem is solved. However, more importantly, research into the mechanisms of combination therapy is insufficient to offer guidance for clinical research.

## 6. Safety and Toxicity

The world’s first clinical trial of siRNA-based therapy was conduct in 2004. To date, the long-term safety and toxicity data of siRNA therapeutics are still being studied, and both siRNA and nano-delivery systems need to be considered. Infusion-related reactions (IRRs) are common adverse reactions (ADR) of NPs [180]. For example, mild to moderate ADR was observed during the clinical translation of Patisiran^®^, and the main symptoms included back pain, body aches or pain, chills, headache, nausea and sore throat [181]. These symptoms can be alleviated by slowing down the infusion rate or prevented by using dexamethasone, acetaminophen and H2 blockers. In addition to silencing target genes, siRNA may also knock down unwanted genes [182]. Off-target effects of siRNA cause unanticipated side effects, which makes the treatment more complicated. Alnylam showed that that the hepatotoxicity of GalNAc-siRNA conjugates is caused by off-target effects [183]. Although glycol nucleic acid (GNA) modification of siRNA may potentially reduce the off-target effects of GalNAc-siRNA conjugates, there is currently a lack of preclinical animal models to accurately predict the side effects due to sequence-specific RNAi-based mechanism in different animals.

Published clinical trials report that the COVID-19 mRNA vaccine based on LNP delivery system has high efficacy rates of 94–95%, limited side effects and low incidence of adverse reactions. However, some populations around the world may still reject the mRNA vaccine due to its rapid pace of development, emergency approval, and uncertainty about potential long-term adverse effects. However, siRNA-based cancer therapeutics are fundamentally different from the COVID-19 mRNA vaccine in terms of the acceptability of patients because: (1) the vaccine is a preventive measure, while the siRNA drug is a therapeutic modality, and (2) vaccines target healthy populations who may be infected with SARS-CoV-2, while siRNA drugs target patients. People think more about risks than benefits when it comes to preventing rather than treating disease. When faced with survival, cancer patients may not first consider the potential side effects of LNP in immune stimulation.

## 7. Outlook

Although siRNA-based cancer therapeutics have proved to be promising in preclinical studies and clinical trials, several key challenges need to be addressed before moving into final clinical applications in cancer patients.

Firstly, the inherent instability of siRNA that makes it susceptible to nucleases and cause it to degrade quickly in vivo has limited its in vivo application. To address this problem, chemical modification of siRNA has been extensively studied. As mentioned above, the stability of siRNA can be improved by a series of chemical modifications on its backbone. Secondly, effective extra-hepatic tissues/cells targeting to limit the widespread use of siRNA therapeutics in cancer treatment has always been difficult. Advances in drug delivery technology are expected to improve this limitation. In addition to the EPR effect, an active targeting strategy is also utilized to enhance specific cell uptake. For example, modification with specific targeting ligands, such as the prostate-specific membrane antigen (PSMA) ligand, has proven to promote the cellular uptake of NPs at the site of prostate cancer, and is already in phase II clinical trials [184]. Thirdly, the development of multifunctional delivery systems that can meet the needs of diverse treatment modalities is the main direction of cancer nanomedicine in the future. However, the selection of gene targets and treatment methods, as well as the methods of synergistic combination therapy, need to be tailored to local conditions. Although the above combined strategies have brought high effectiveness, challenges such as complex material composition may promote instability and hinder large-scale production. The preparation of delivery system for clinical application should be simple and reproducible. Additionally, beyond the instability and specific targeting issues, the main difficulty in limiting the action of RNAi is effective endosomal escape. To further enhance the activity of RNAi, more efforts need to be made in endosomal escape, such as the development of safe and effective endosomal escape agents. Finally, although many siRNA drugs have been found to be safe, effective and well-tolerated in phase I or II trials, few have been successful translated into clinical use. Currently, there is a lack of suitable preclinical models to evaluate the therapeutic outcomes of siRNA drugs, so it is necessary to establish animal models that can better simulate the human environment. In addition, the selection of target patient populations is also crucial for successful clinical translation, as the right population may have greater therapeutic benefits. Therefore, in order to improve the success rate in the clinical translation of siRNA-based cancer therapeutics, the following issues should be considered: (1) the biosafety and biodegradability of siRNA delivery vehicles; (2) simple, efficient, controlled and reproducible protocols of scale-up production for clinical trials and post-marketing assessment; (3) the enhancement of targeting specificity and endosomal escape capability of nanocarriers to tumor tissues/cells; (4) the selection of appropriate preclinical animal models that can accurately predict the clinical PK and PD of siRNA drugs; (5) the in vivo safety profiles of NP-based siRNA therapeutics, including undesirable cytotoxicity and immune stimulation; (6) accurate stratification of different tumor patient populations, and (7) in combination with other therapeutics, the dose and sequence of administration should be considered. These non-trivial challenges can be potentially addressed by interdisciplinary approaches in the fields of chemistry, materials, biology and medicine.

## 8. Conclusions

With the FDA approval of Patisiran^®^, more siRNA therapeutic drugs are undergoing a translation from research to the clinic. However, to date, none of the siRNA therapeutics have been approved for cancer treatment, indicating that there are still significant challenges and opportunities in this field. As demonstrated in preclinical studies and clinical trials, there is no doubt about the huge gene silencing efficiency of siRNA and its potential curative effect on cancer. One of the key challenges for the clinical translation of siRNA-based cancer therapeutics is to overcome all the extracellular and intracellular barriers, and successfully delivery siRNA into the cytoplasm of cancer cells. With the collaborative efforts of academia and industry, nanotechnology-based delivery strategies are expected to achieve selective delivery of siRNA into extrahepatic cancer cells and promote the clinical application of siRNA cancer therapeutics. With the rapid progress of tumor biology, siRNA therapeutics will ultimately become an effective means of cancer treatment through the rational design of target genes and combination with other therapeutic strategies. Although new CRISPR/Cas9-based gene editing technologies are emerging, RNAi-based technology seems safer because of its transient and reversible gene silencing, without permanent genetic alterations. In conclusion, as a new class of anti-cancer therapeutics, siRNA drugs delivered by nanoparticles show great promise in cancer therapy and are expected to enter clinical practice in the coming years.

## Figures and Tables

**Table 1:** Currently approved siRNA therapeutics for non-cancerous diseases.

Name (Market Name)	Company	Gene Targets/Indications	Organ	Route of Administration	Delivery System	Approval
Patisiran (Onpattro)	Alnylam	TTR/hereditary transthyretin amyloidosis (hATTR)	Liver	Intravenous	LNP	August 2018 (FDA)
Givosiran (Givlaari)	Alnylam	ALAS1/acute hepatic porphyria (AHP)	Liver	Subcutaneous	GalNAc conjugate	November 2019 (FDA)
Lumasiran (Oxlumo)	Alnylam	HAO1/primary hyperoxaluria type 1 (PH1)	Liver	Subcutaneous	ESC-GalNAc conjugate	November 2020 (FDA, EMA)
Inclisiran (Leqvio)	Novartis	PCSK9/hyperlipidemia	Liver	Subcutaneous	ESC-GalNAc conjugate	2020 (EMA)

**Table 2:** siRNA-based cancer therapeutics in clinical trials.

Name	Indications (Tumor Types)	Delivery System	Gene Targets	Sponsor	Phase	Status	NCT ID
SiRNA-EphA2	Advanced Malignant Solid Neoplasm	Neutral liposome	EphA2	M.D. Anderson Cancer Center	I	Active, not recruiting	NCT01591356
iExosomes	Pancreatic Cancer	Exosomes	KRAS G12D	M.D. Anderson Cancer Center	I	Recruiting	NCT03608631
Atu027 Atu027 +Gemcitabine	Advanced Solid Tumors Advanced/metastatic pancreatic cancer	Lipople xLipoplex	PKN3 PKN3	Silence Therapeutics GmbH Silence Therapeutics GmbH	I II	Completed Completed	NCT00938574 NCT01808638
CALAA-01	Solid Tumors	Transferrin receptor-targeted cyclodextrin nanoparticle	RRM2	Calando Pharmaceuticals	I	Terminated	NCT00689065
ALN-VSP02	Solid Tumors	lipid nanoparticle	VEGF, KSP	Alnylam Pharmaceuticals	I	Completed	NCT00882180
siG12D-LODER	Pancreatic Ductal Adenocarcinoma	Miniature biodegradable polymeric matrix	KRAS G12D	Silenseed Ltd.	II	Unknown	NCT01676259
TKM-080301	Advanced Solid Tumors	SNALP	PLK1	Arbutus Biopharma Corporation	II	Completed	NCT01262235 NCT02191878
STP705	Squamous Cell Carcinoma in Situ	Peptide-Nano particle	TGF-β1, COX-2	Sirnaomics	II	Recruiting	NCT04844983
NBF-006	Non-Small Cell Lung Cancer Pancreatic Cancer Colorectal Cancer	LNP	GSTP	Nitto BioPharma	I	Recruiting	NCT03819387
DCR-MYC	Hepatocellular Carcinoma	EnCore LNP	MYC	Dicerna pharmaceuticals	1b/2	Terminated	NCT02314052

EphA2: ephrin A2 receptor; KRAS: kristen rat sarcoma viral oncogene homolog; PKN3: protein kinase N3; RRM2: M2 subunit of ribonucleotide reductase; VEGF: vascular endothelial growth factor; KSP: kinesin spindle protein; PLK1: polo-like kinase 1; TGF-β1: transforming growth factor beta 1; COX-2: cyclooxygenases 2; GSTP: glutathione S-transferase P; Cited from http://www.clinicaltrials.gov (accessed on 12 July 2022).

## Data Availability

The data in Table 2 from http://www.clinicaltrials.gov (accessed on 12 July 2022).
